# Innate Immune Signaling and Proteolytic Pathways in the Resolution or Exacerbation of SARS-CoV-2 in Covid-19: Key Therapeutic Targets?

**DOI:** 10.3389/fimmu.2020.01229

**Published:** 2020-05-28

**Authors:** Jean-Michel Sallenave, Loïc Guillot

**Affiliations:** ^1^INSERM UMR1152, Laboratoire d'Excellence Inflamex, Faculté de Médecine, Hôpital Bichat, Université de Paris, Paris, France; ^2^Sorbonne Université, INSERM UMR S 938, Centre de Recherche Saint-Antoine (CRSA), Paris, France

**Keywords:** COVID-19, SARS-CoV-2, coronavirus, protease, lung innate immunity

## Abstract

COVID-19 is caused by the Severe Acute Respiratory Syndrome (SARS) coronavirus (Cov)-2, an enveloped virus with a positive-polarity, single-stranded RNA genome. The initial outbreak of the pandemic began in December 2019, and it is affecting the human health of the global community. In common with previous pandemics (Influenza H1N1 and SARS-CoV) and the epidemics of Middle east respiratory syndrome (MERS)-CoV, CoVs target bronchial and alveolar epithelial cells. Virus protein ligands (e.g., haemagglutinin or trimeric spike glycoprotein for Influenza and CoV, respectively) interact with cellular receptors, such as (depending on the virus) either sialic acids, Dipeptidyl peptidase 4 (DPP4), or angiotensin-converting enzyme 2 (ACE2). Host proteases, e.g., cathepsins, furin, or members of the type II transmembrane serine proteases (TTSP) family, such as Transmembrane protease serine 2 (TMPRSS2), are involved in virus entry by proteolytically activating virus ligands. Also involved are Toll Like Receptor (TLR) family members, which upregulate anti-viral and pro-inflammatory mediators [interleukin (IL)-6 and IL-8 and type I and type III Interferons among others], through the activation of Nuclear Factor (NF)-kB. When these events (virus cellular entry and innate immune responses) are uncontrolled, a deleterious systemic response is sometimes encountered in infected patients, leading to the well-described “cytokine storm” and an ensuing multiple organ failure promoted by a downregulation of dendritic cell, macrophage, and T-cell function. We aim to describe how the lung and systemic host innate immune responses affect survival either positively, through downregulating initial viral load, or negatively, by triggering uncontrolled inflammation. An emphasis will be put on host cellular signaling pathways and proteases involved with a view on tackling these therapeutically.

## Introduction

COVID-19 is a respiratory disease whose aetiologic agent is a novel beta coronavirus (CoV) called Severe Acute Respiratory Syndrome (SARS)-CoV-2/2019-nCov. The initial outbreak of the pandemic began in December 2019, and it is currently affecting the health and safety of the global community. Indeed, on May 12, 2020, 4.5 million worldwide cases were confirmed (probably a significant under-estimation given the number of untested asymptomatic subjects), with a death toll exceeding 286,000. Before the SARS-CoV-2 outbreak, two related highly pathogenic CoVs viruses, Middle east respiratory syndrome (MERS)-CoV (1) and SARS-CoV (2), provoked catastrophic epidemics and pandemics, respectively. Unfortunately, no drugs nor vaccines have currently been approved to prevent or treat these viral episodes. The first anatomical/histological reports from the lungs of severely SARS-CoV-2-affected patients experiencing acute respiratory disease syndrome (ARDS) revealed excessive inflammatory activation and destruction of the bronchial and alveolar epithelium, features already observed during the first SARS pandemics in 2003 (3, 4). Indeed, in the latter pandemic, lung alveolar epithelial cells were identified as the most likely site of virus replication, and it was suggested that alveolar macrophages may be responsible for the dissemination of viruses within the lungs (3). In accordance, initial histological analyses of lung biopsies from patients positive for SARS-CoV-2 have shown exfoliation of the bronchial epithelium, which may induce altered mucociliary clearance and affect host immune responses (5).

Indeed, there is no doubt that the latter are involved in modulating disease onset and progression. For example, early studies report that, similarly with what was observed with SARS-CoV, lymphopenia [sometimes equivalent or more severe than that observed in human immunodeficiency virus (HIV) infection] is often observed in severely affected patients progressing to ARDS. Despite, or maybe correlated with this, aberrant non-effective innate immune host responses seem associated with severe lung disease during SARS (6–12).

The following sections will give an overview of the molecular and cellular mechanisms underpinning SARS-CoV virus infections and how lung and systemic host innate immune responses affect survival either positively, through downregulating the initial viral load, or negatively, by triggering uncontrolled inflammation. A particular emphasis will be put on the description of the host cellular signaling pathways and proteases involved with a view on tackling these therapeutically.

## Mechanisms of Entry of Coronaviruses into Target Epithelial Cells (SEE FIGURE 1A)

CoVs are enveloped viruses with a positive-polarity, single-stranded RNA genome encoding four structural proteins: the transmembrane trimeric spike glycoprotein (S, composed of two subunits S1 and S2), envelop (E), matrix (M), and nucleocapsid (N) (13).

**Figure 1:**
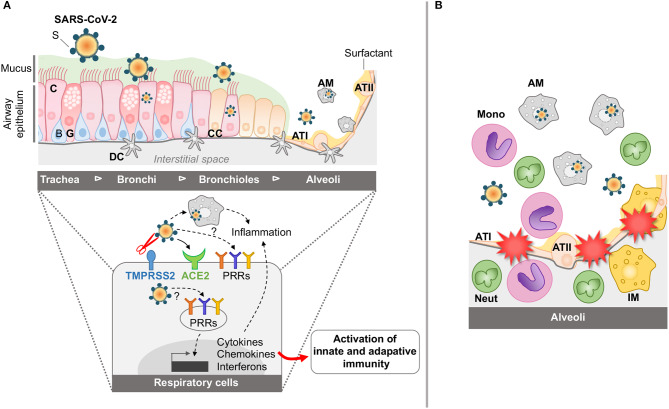
Schematic representation of airway and lung infection by SARS-CoV-2 at early time points **(A)** and during SARS **(B)**. **(A)** The airway epithelium is composed of various cell types including ciliated (C), basal (B), glandular (G), and club cells (CC). It is covered by mucus (M) involved in the mucociliary clearance. Distal to the lung, the alveoli include alveolar type I (ATI) and type II (ATII) cells coated with surfactant (S). The airways are also protected by the resident alveolar macrophages (AM) and dendritic cells (DC). Through the action of host proteases, including the TTSP Transmembrane protease, serine 2 (TMPRSS2), and the interaction of SARS-CoV-2 with its cellular receptor angiotensin-converting enzyme 2 (ACE2), SARS-CoV-2 can enter and replicate into airway epithelial cells and AM. Also, interaction of the SARS-CoV-2 with the PRRs is instrumental in inducing cytokine, chemokine, and interferon responses for the establishment of innate and adaptive immune responses. **(B)** During SARS, innate immune responses are exacerbated in the alveolar space, with accumulation of activated monocytes (mono), activated macrophages (AM), interstitial macrophages (IM), and neutrophils (neut), leading to dysregulated inflammation, disruption of the alveolar-capillary membrane and tissue damage.

The entry of CoV viruses into host epithelial cells is mediated by the interaction between the viral envelope S protein homotrimers and the cell surface receptors. Following proteolytic cleavage of the CoV S protein (“priming”), the S1 ecto-domain recognizes a membrane receptor [angiotensin-converting enzyme 2 (ACE2) for SARS-Cov and SARS-Cov-2 as well as Dipeptidyl peptidase 4 (DPP4) for MERS-Cov], whereas the S2 C-terminal domain is involved in cell fusion and viral entry (14–16). This mechanism of action is very similar to that used by *Influenza*, except that the latter use sialic acids as the cognate receptor for its hemagglutinin (HA) ligand. Importantly, many viruses (*Influenza*, MERS, CoV, and Paramyxoviruses such as Hendra and Nipah viruses) use similar host proteolytic enzymes for cleaving their ligands (HA and S), namely, mostly lysosomal (Cathepsins B, L), furin, or trypsin-like proteases (17, 18). Indeed, it is believed that it is the cellular source of these proteases that may determine the infectivity spectrum of these viruses, with the lung and the gastro-intestinal tract being high producers (19, 20).

Although a variety of these proteases have been studied and shown to be involved to varying degrees in virus activation, including neutrophil elastase (21), proteases of the type II transmembrane serine proteases (TTSP) family [HAT, Transmembrane protease, serine (TMPRSS)2, and TMPRSS4] have recently been demonstrated to be particularly important, albeit probably at different stages of the virus cell cycle (19, 20, 22, 23). In particular, recent research on SARS-Cov-2 has focused on TMPRSS2 and has shown it to be important (although mostly using cell lines infected with pseudotyped virus particles bearing SARS-Cov-2 S protein) for virus entry (24, 25). In that context, it has also been demonstrated that the serine protease inhibitor camostat (see also below section on Therapeutic targets and Conclusion) was protective (24, 25). In contrast, DPP4 which is necessary for the entry of MERS-CoV (26, 27) is not involved in SARS-Cov-2 entry (24). Unlike other SARS-CoVs, the S protein of SARS-CoV-2 has a furin cleavage site at the boundary between the S1 and S2 subunits, which is processed during biogenesis and which may explain CoV-2 high infectivity (28). Although mechanistic studies are obviously still in their infancy, it is very likely that SARS-CoV and SARS-CoV-2 target mainly respiratory epithelial cells with similar mechanisms. Indeed, as indicated above, initial work has shown that ACE2 is the S receptor for both SARS-CoV (29) and SARS-CoV-2 viruses (24, 28, 30), and structural studies using cryo-electron microscopy suggest a binding of two S protein trimer to an ACE2 dimer (28, 30).

Whether this is strictly dependent on ACE2/protease expression is debatable since ACE2 is present in other tissues in humans [such as the intestine, kidney, and testis (31)]. Indeed, “seasonal” low pathogenic CoVs (e.g., CoV-229E, CoV-OC43) infect mostly upper airways, whereas pathogenic CoVs (SARS-CoV/SARS-CoV-2 and MERS) have a tropism for the distal lung and can cause severe pneumonia and ARDS (32), as currently demonstrated again in the present pandemic. Indeed, potentially explaining this is the fact that seasonal coronaviruses do not use ACE2 as a receptor. *In vitro*, primary nasal and tracheobronchial epithelial cells as well as the Calu-3 bronchial cell line were shown to express ACE2 (the latter not colocalizing with cilia), and their infection with SARS-CoV was shown to be highly cytotoxic (33, 34). In the distal lung, as hinted above, primary alveolar type II epithelial (ATII) cells are also permissive to SARS-CoV infection (35, 36). SARS-CoV-2 has also been shown to infect various respiratory epithelial cell lines including A549 (alveolar origin), BEAS2-B (bronchial origin), Calu-3 cells, as well as primary human bronchial epithelial cells (24). Besides the lung, ACE2 is also highly expressed in the intestine (37), and gastrointestinal symptoms have been recorded with COVID-19 (38). It was shown that SARS-COV2 is able to infect enterocytes as well as intestinal organoids and induces a viral response characterized by the expression of mediators related to type I and III IFN (39).

Even if SARS-CoV2 is thought to originate from bats, the intermediate host between bats and humans is still unknown. SARS-CoV was previously shown to infect various wild and domestic animals, including cats, ferrets and pigs (40–42). Similarly, recent work reveals that domestic animals, including ferrets and cats, are permissive to SARS-CoV-2 infection. In contrast, the virus replicates poorly in pigs, ducks, chickens, and dogs (43).

Given the described importance of host proteases in mediating infectivity of a number of viruses, it is no surprise that, upon virus infection, murine knock-out (KO) for some of these molecules has shown some protection. For example, TMPRSS2-KO mice were protected from pulmonary disease and death following H1N1 and H7N9 *Influenza* infection, but not from that of the influenza H3N2 subtype, demonstrating some specificity and showing also that other TTSP proteases [such as DESC1 (TMPRSS11E) and MSPL (TMPRSS13)] or other factors may be important (44–47).

Similarly, TMPRSS2 KO mice showed reduced body weight and viral loads compared to WT mice in animals infected with SARS-CoV (48).

Also, it was demonstrated that over-expression of the human DPP4 in mice promoted MERS-CoV infection, causing lethal disease (49), and that TMPRSS2 was instrumental in that context (48).

## Activation/Modulation of Host Signaling Pathways (SEE FIGURE 1A)

### Epithelial Cells

The control of viral infection requires an optimal and innate coordinated host antiviral immunity. This response is activated by various sensors, including pattern recognition receptors (PRR), which recognize pathogen-associated molecular patterns (PAMPs). Although for many viruses, viral RNA is a PAMP classically detected by different sensors, including Toll-Like Receptors (TLR)3 (which senses double stranded (ds)RNA), TLR7 and TLR8 [which sense single stranded (ss)RNA], RIG-I (which senses short dsRNA and ssRNA specific motifs), and MDA-5 (which senses long dsRNA) (50), the sensors potentially recognizing SARS-CoV genomic material are still elusive. In addition, although, as mentioned above, distal peripheral lung alveolar epithelial cells seem to harbor SARS-CoV infection *in vivo*, and although respiratory epithelial cells are known to express TLR3, TLR7, and TLR8 (51, 52) and initiate innate immunity in the lung (53), the study of these cells in anti-CoV responses has been hampered by their general poor permissibility to the virus *in vitro* (except for intestinal Caco-2 and HEK293 kidney epithelial cells) (54). In that respect, although the specific PRR involved was not identified, the M protein of SARS-CoV was indeed shown to induce interferon (IFN)-β in a TLR-related-TRAF3-independent mechanism in HEK293 cells (55). Regarding the lung, the differentiated Calu-3 cell line [when cultured at the air-liquid interface (ALI)] is the model of choice: in that set-up, SARS-CoV infection triggered an inflammatory response characterized by increased production of interleukin (IL)-6, IL-8, gamma interferon (IFN-γ), inducible protein 10 (IP-10), and activation of the transcription factor NF-κB (56). However, the kinetics of this response was extremely slow, and importantly, type I IFN, an important mediator of anti-viral responses, was undetected.

Also, another study involving A549 cells demonstrated that the trimeric spike S glyprotein and virus-like particles were able to modestly upregulate CCL2, an important monocytic chemokine (57).

In addition to lung epithelial cells cultured at ALI, precision-cut lung slices could also be an interesting tool to study SARS-CoV2-cells interactions (58), as demonstrated in Influenza infections with human (59) or animal-derived material (60).

As mentioned above, TTSPs can activate virus-ligands (HA and S protein), but they are also able to modulate cell signaling pathways. For example, recombinant HAT is able to activate mucin gene expression in NCI-H292 lung epithelial cells (61). Relatedly, we have shown both *in vitro* in epithelial cells and in a murine model that *Influenza H3N2* is able to upregulate mucin expression and that this is dependent on human (or mouse) HAT upregulation and TACE activity (62). Interestingly, Haga et al. have shown that inhibiting TACE prevents SARS-CoV cellular entry (63). Strengthening the signaling potential of the receptors, Iwata-Yoshikawa et al. demonstrated *in vivo* that poly IC (TLR3 ligand) induces the expression of a variety of pro-inflammatory mediators (CCL2, KC, and IL-1) through the expression of TMPRSS2 (48).

In addition, although unclear as whether it is beneficial or detrimental to the host cell, SARS-CoV have been shown to activate host stress response, apoptosis, and autophagy (13). These are also various pathways that may also need to be evaluated therapeutically in the context of the current pandemic. Relatedly, we have shown that chloroquine, which also inhibits the autophagic cellular flux by decreasing autophagosome-lysosome fusion, can inhibit Influenza-mediated CCL5 production (64).

Importantly, after having established a foothold in the epithelial compartment, SARS-CoV can disrupt the epithelial polarity, thereby getting access to the parenchyma tissue: for example, it has been shown that the virus membrane protein E binds to PALS1 (Protein Associated With Lin Seven 1), a junction protein involved in epithelial polarity, and modifies its cellular distribution at the surface of HEK-293 cells (65).

### Myeloid Cells and Myeloid-Epithelial Cells Interaction

Myeloid cells, e.g., alveolar and interstitial macrophages or dendritic cells (DCs), elicit different immune responses toward influenza viruses, according to their subtypes (66). It is thus predictable that specificities may also exist with respect to SARS-COV-2 infections. Indeed, although studies are scant, these cells have generally been shown to be poorly permissive to SARS-CoV replication (54, 67, 68).

However, a few studies have shown that myeloid cells can respond to SARS-Cov infection. Indeed, Dosch et al. showed that the S protein could, through TLR2, trigger NF-κB activation and inflammatory responses in peripheral blood mononuclear cells (PBMC) (69). Also, in common with epithelial cells, it was shown that PBMCs and DCs infected with SARS-CoV produced cytokines and chemokines such as and C-C Motif Chemokine Ligand (CCL)-2 and/or C-X-C Motif Chemokine Ligand (CXCL)-10/RANTES/Tumor Necrosis Factor (TNF)/IL-8/IL-6, but, importantly, not IFN-β (67, 68). By contrast, a study performed mostly on THP-1 macrophages suggest that MERS S protein suppresses macrophages pro-inflammatory responses through DPP4-induction of IRAK-M and PPARγ (70).

Furthermore, in an interesting “2-way” system involving differentiated SARS-permissive lung Calu-3 cells and monocyte-derived Macs and DCs, it was shown that mediators produced by Calu-3 cells activate cytokine production by macrophages (IL-1β, G-CSF, MIP-1, and TNF-α) and DCs (IL-12p40, MIP-1, IFN-γ, IL-6, IL-8, and MCP-1) but that some of these Calu-3 derived mediators (in particular IL-6 and IL-8) compromised the ability of DCs and Macs to activate naïve T cells and phagocytosis (4, 56). This echoes data obtained from patients suggesting that SARS may in fact be partly caused by a “paralysis” of the adaptive immune system, characterized by a diminished number of immune cell types including T lymphocytes, DCs and Macs (4).

### From Murine Models to Human Genetics

Demonstrating that SARS-CoV can induce TLR-dependent host responses *in vivo, Tlr4, Tlr3*, and *Tram* KO mice were shown to be more susceptible to mouse-adapted SARS-CoV, albeit without exhibiting extra mortality (71). In comparison, mice deficient for the signaling molecule *Trif* were highly susceptible to CoV infections, exhibited diminished lung function, aberrant inflammatory responses, and importantly, higher mortality (71).

In addition, a mouse genetic study revealed that the TLR adaptor protein *Ticam2* was a susceptibility gene to SARS-CoV (72); mice KO for *Ticam2* (72), but also *MyD88* (73), another TLR adaptor protein, were highly susceptible to a mouse-adapted SARS-CoV lung infection. Since polymorphisms of TLRs and MyD88 have been associated in humans with heightened sensitivity to a variety of pathogens (74), these studies, in addition to demonstrating the role of TLR pathways in the SARS-CoV infection, suggested a human genetic predisposition to SARS-CoV, and this could explain the variability of severity in patients with COVID-19 disease. Forthcoming human genetic studies from international collaborative efforts (https://www.covid19hg.org) could reveal genetic variants associated with SARS-CoV2 susceptibility, as in the gene encoding ACE2 as recently suggested (75). Indeed, *ACE2* genetic variants may be associated with a modulated ACE2 protein expression, the SARS-Cov-2 receptor, which may explain in part patients' susceptibility to infection. Genes associated with TLR pathways also represent good candidates, as demonstrated in other respiratory viral infection (e.g., influenza) where *TLR3* variants (76) were shown to modulate its virulence.

### Maladaptive Activation of Innate Immune Responses (see Figure 1B)

As already mentioned above, aberrant maladaptive innate immune host responses, including “cytokine storm” events, have been associated with severe lung disease and the development of ARDS during SARS and the COVID-19 current episode. Mechanistically, these events usually occur at a late stage of the disease, and several mechanisms have been proposed. In particular, a murine study has shown that a prolonged (albeit delayed, as demonstrated also *in vitro*, see above) type I IFN signaling was instrumental in triggering over-exuberant innate inflammatory monocytes–macrophages immune responses and an impaired virus-specific T-cell response (77).

In complement to the mechanism proposed above, increased lung inflammatory protease (neutrophil elastase and metalloprotease) activity has been demonstrated in ARDS (78, 79), with a concomitant imbalance between protease and protease inhibitors activity (80). In addition, although not yet measured, to our knowledge, in SARS murine models, we and others have shown increased protease-mediated lung damage in mice infected with *Influenza* (81–83). Additionally, in a MERS-CoV murine model, it was shown that excessive complement activation was partly responsible for exacerbated lung inflammation (84).

Lastly, “cytokines storm” may also results from SOCS (suppressors of cytokine signaling) inhibition (85). Indeed, upon Influenza infection, SOCS1 and SOCS3 were shown to reduce type I IFN antiviral responses in human bronchial epithelial cells (86). Also, SOCS4-deficient mice exhibited heightened sensitivity to Influenza infection (87). Studies about SOCS involvement during coronavirus infections are currently lacking and should therefore bring new interesting information.

## Potential Therapeutic Targets and Conclusion (See FIGURE 2)

On May 12, 2020, using the term “COVID,” an unbiased search of already registered trials on https://clinicaltrials.gov/ retrieved 1,409 hits, and, when refined with “double blind/placebo,” 119 hits were found. Although the number of trials that are ongoing or “under recruitment” is expectedly very high, the range of molecules tested is relatively narrow and aimed at targeting mainly antivirals. These include remdesivir (21 hits), lopinavir/ritonavir (also used in AIDS), as well as interferons (46 hits). Also falling in that category are trials testing molecules aiming to block viral entry at the cellular surface by targeting ACE-inhibitors (32 hits) or the membrane proteases of the TTSP family (see above) using camostat mesilate (5 hits). Repurposing of non-antiviral drugs may offer new promising options, such as with Ivermectin—an FDA-approved anti-parasitic drug widely available and recently shown to inhibit SARS-CoV-2 *in vitro* (88).

**Figure 2:**
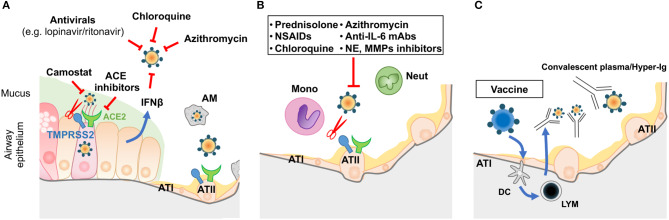
Prophylactic/therapeutic approaches to COVID-19. **(A)** Potential therapeutic anti-viral approaches during the early phase of infection. **(B)** Potential anti-inflammatory strategies targeting the alveolar space during the SARS period of COVID disease. **(C)** Immune intervention (vaccination of the population prior to epidemic episodes) or use of convalescent plasma/hyper-immune globulins on infected patients. LYM: lymphocytes; other acronyms are as described in Figure 1.

Because the virus load is not necessarily correlated with symptoms deterioration in SARS (the latter being often caused by worsening of inflammation at day 7–10 post onset of clinical signs), it follows that anti-inflammatory drugs could/should be prescribed during that stage of the disease (8).

In that context, “classical” anti-inflammatory drugs are indeed currently being tested against COVID-19 [e.g., methylprednisolone, budesonide, hydrocortisone, azithromycin, and non-steroïdal anti-inflammatory drugs (NSAIDs)]. In addition, more specific agents are also being investigated, targeting either IL-β (anakinra, 13 hits), IL-6 signaling (Siltuximab/3 hits, Tocilizumab/42 hits, Sarilumab/13 hits), or CD24 (CD24Fc) with the main objective to modulate the “cytokine storm.”

However, chloroquine/hydroxychloroquine has, so far, undoubtedly taken the lion's share (178 hits), and it has attracted a lot of media attention. In that respect, the results from an initial pan-European endeavor (“Discovery”), now conducted largely in France because of enrollment difficulties, are eagerly awaited. This drug has a “mixed” mode of action. Indeed, it acts as an anti-viral (presumably through inhibition of lysosomal enzymes requiring an acidic pH and of activation of endolysosomes, see above section “Mechanisms of entry”) and as an anti-inflammatory molecule, and it has notably been used in inflammatory rheumatic diseases (89). Despite a relative safe profile, having been administered to millions of people over the years, worries have nevertheless arisen about cardiac issues in many individuals with severe Covid-19, and this will have to be properly assessed (90).

Regardless, the ultimate prize in the fight against COVID-19 (or further SARS-CoV infections) undoubtedly lies with the future generation of effective vaccines and the development of neutralizing antibodies (91, 92).

Unfortunately, coronavirus vaccines in general have attracted less attention compared to the effort dedicated to vaccines against other potential pandemic viruses such as Influenza. For example, from 2012 onwards, few SARS-CoV vaccines reached phase 1 clinical trials for lack of interest from the pharmaceutical industry when it became evident that the virus was not making a “comeback” after its initial appearance. However, although probably too late for affecting the current “first wave” of SARS-CoV-2 pandemic, many pharmaceutical companies and research laboratories are now working on a plethora of vaccine formulations [for a review, see (91) and https://clinicaltrials.gov, the latter reporting so far 83 clinical trials on vaccines].

Indeed, in pre-clinical studies, the determination of cryo-EM structures of the SARS-CoV-2 S ectodomain trimer is providing a blueprint for the design of vaccines and inhibitors of viral entry (28). In this context, promising results show that murine polyclonal antibodies against S protein of SARS-CoV are able to elicit polyclonal antibody responses, preventing SARS-CoV-2 entry into cells, and thus indicating that cross-neutralizing antibodies targeting conserved S epitopes can be elicited upon vaccination (28).

In addition to testing the best SARS-CoV-2 specific epitopes from the most suitable proteins (S, N, etc.) and way of administration (best vectors, etc.), it is important to select the best animal models. Although convincing murine studies are still pending, as indicated above in the section “Mechanisms of entry…”, studies in other animals investigated the virus susceptibility of chickens, ducks, dogs, pigs, cats, and ferrets, with the latter two being the most permissive (43). Further up in the phylogenetic scale, a recent study reported that an inactivated vaccine candidate for SARS-CoV-2 was protective in macaques (93).

Finally, large epidemiological studies have demonstrated that Bacille Calmette-Guerin (BCG) can heterologously protect against virus infections [e.g., yellow fever virus (94), probably by tapping on trained immunity mechanisms (95, 96)]. Using such adjuvant-mediated strategies against SARS-CoV viruses may therefore be an exciting avenue worthwhile pursuing (97–99).

## Author Contributions

All authors listed have made a substantial, direct and intellectual contribution to the work, and approved it for publication.

## Conflict of Interest

The authors declare that the research was conducted in the absence of any commercial or financial relationships that could be construed as a potential conflict of interest.

## Abbreviations

CoV: Coronavirus
SARS: Severe Acute Respiratory Syndrome
MERS: Middle east respiratory syndrome
ARDS: acute respiratory disease syndrome
HIV: human immunodeficiency virus
S: Spike
E: envelop
M: matrix
N: nucleocapsid
HA: hemagglutinin
ACE2: angiotensin-converting enzyme 2
DPP4: Dipeptidyl peptidase 4
TTSP: type II transmembrane serine proteases
TMPRSS: Transmembrane protease serine
NF-kB: Nuclear Factor
IL: interleukin
KO: knock-out
ATI: alveolar type I epithelial cell
ATII: alveolar type II epithelial cell
PRR: pattern recognition receptors
PAMPs: pathogen-associated molecular patterns
TLR: Toll-Like Receptors
IFN: interferon
Mac: macrophages
DCs: dendritic cells
PBMC: peripheral blood mononuclear cells
CCL2: C-C Motif Chemokine Ligand 2
C: ciliated
B: basal
G: glandular
CC: club cells
M: mucus
AM: alveolar macrophage
BCG: Bacille Calmette-Guerin.

## References

[1] Chafekar A Fielding BC. MERS-CoV: understanding the latest human coronavirus threat. Viruses. (2018) 10:93. 10.20944/preprints201711.0198.v2 29495250 PMC5850400

[2] Peiris JS Yuen KY Osterhaus AD Stohr K. The severe acute respiratory syndrome. N Engl J Med. (2003) 349:2431–41. 10.1056/NEJMra032498 14681510

[3] Nicholls JM Poon LL Lee KC Ng WF Lai ST Leung CY. Lung pathology of fatal severe acute respiratory syndrome. Lancet. (2003) 361:1773–8. 10.1016/S0140-6736(03)13413-7 12781536 PMC7112492

[4] Gu J Gong E Zhang B Zheng J Gao Z Zhong Y. Multiple organ infection and the pathogenesis of SARS. J Exp Med. (2005) 202:415–24. 10.1084/jem.20050828 16043521 PMC2213088

[5] Yao XH Li TY He ZC Ping YF Liu HW Yu SC. [A pathological report of three COVID-19 cases by minimally invasive autopsies]. Zhonghua Bing Li Xue Za Zhi. (2020) 49:411–17. 10.3760/cma.j.cn112151-20200312-00193 32172546

[6] Chen G Wu D Guo W Cao Y Huang D Wang H. Clinical and immunologic features in severe and moderate Coronavirus Disease 2019. J Clin Invest. (2020) 130:2620–9. 10.1172/JCI137244 32217835 PMC7190990

[7] Giamarellos-Bourboulis EJ Netea MG Rovina N Akinosoglou K Antoniadou A Antonakos N. Complex immune dysregulation in COVID-19 patients with severe respiratory failure. Cell Host Microbe. (2020) 10.1016/j.chom.2020.04.009. [Epub ahead of print]. 32320677 PMC7172841

[8] Huang C Wang Y Li X Ren L Zhao J Hu Y. Clinical features of patients infected with 2019 novel coronavirus in Wuhan, China. Lancet. (2020) 395:497–506. 10.1016/S0140-6736(20)30183-5 31986264 PMC7159299

[9] Lescure FX Bouadma L Nguyen D Parisey M Wicky PH Behillil S. Clinical and virological data of the first cases of COVID-19 in Europe: a case series. Lancet Infect Dis. (2020) 20:697–706. 10.1016/S1473-3099(20)30200-0 32224310 PMC7156120

[10] Thevarajan I Nguyen THO Koutsakos M Druce J Caly L van de Sandt CE. Breadth of concomitant immune responses prior to patient recovery: a case report of non-severe COVID-19. Nat. Med. (2020) 26:453–5. 10.1038/s41591-020-0819-2 32284614 PMC7095036

[11] Wu C Chen X Cai Y Xia J Zhou X Xu S. Risk factors associated with acute respiratory distress syndrome and death in patients with Coronavirus Disease 2019 pneumonia in Wuhan, China. JAMA Intern Med. (2020). 10.1001/jamainternmed.2020.0994. [Epub ahead of print]. 32167524 PMC7070509

[12] Zheng HY Zhang M Yang CX Zhang N Wang XC Yang XP. Elevated exhaustion levels and reduced functional diversity of T cells in peripheral blood may predict severe progression in COVID-19 patients. Cell Mol Immunol. (2020) 17:541–3. 10.1038/s41423-020-0401-3 32203186 PMC7091621

[13] Fung TS Liu DX Human coronavirus: host-pathogen interaction. Annu Rev Microbiol. (2019) 73:529–57. 10.1146/annurev-micro-020518-115759 31226023

[14] Millet JK Whittaker GR. Host cell proteases: critical determinants of coronavirus tropism and pathogenesis. Virus Res. (2015) 202:120–34. 10.1016/j.virusres.2014.11.021 25445340 PMC4465284

[15] Tortorici MA Veesler D. Structural insights into coronavirus entry. Adv Virus Res. (2019) 105:93–116. 10.1016/bs.aivir.2019.08.002 31522710 PMC7112261

[16] Coutard B Valle C de Lamballerie X Canard B Seidah NG Decroly E. The spike glycoprotein of the new coronavirus 2019-nCoV contains a furin-like cleavage site absent in CoV of the same clade. Antiviral Res. (2020) 176:104742. 10.1016/j.antiviral.2020.104742 32057769 PMC7114094

[17] Bugge TH Antalis TM Wu Q. Type II transmembrane serine proteases. J Biol Chem. (2009) 284:23177–81. 10.1074/jbc.R109.021006 19487698 PMC2749090

[18] Choi SY Bertram S Glowacka I Park YW Pohlmann S. Type II transmembrane serine proteases in cancer and viral infections. Trends Mol Med. (2009) 15:303–12. 10.1016/j.molmed.2009.05.003 19581128

[19] Bottcher-Friebertshauser E Klenk HD Garten W. Activation of influenza viruses by proteases from host cells and bacteria in the human airway epithelium. Pathog Dis. (2013) 69:87–100. 10.1111/2049-632X.12053 23821437 PMC7108517

[20] Garten W Braden C Arendt A Peitsch C Baron J Lu Y. Influenza virus activating host proteases: identification, localization and inhibitors as potential therapeutics. Eur J Cell Biol. (2015) 94:375–83. 10.1016/j.ejcb.2015.05.013 26095298

[21] Matsuyama S Ujike M Morikawa S Tashiro M Taguchi F. Protease-mediated enhancement of severe acute respiratory syndrome coronavirus infection. Proc Natl Acad Sci USA. (2005) 102:12543–7. 10.1073/pnas.0503203102 16116101 PMC1194915

[22] Bottcher E Matrosovich T Beyerle M Klenk HD Garten W Matrosovich M. Proteolytic activation of influenza viruses by serine proteases TMPRSS2 and HAT from human airway epithelium. J Virol. (2006) 80:9896–8. 10.1128/JVI.01118-06 16973594 PMC1617224

[23] Bertram S Glowacka I Muller MA Lavender H Gnirss K Nehlmeier I. Cleavage and activation of the severe acute respiratory syndrome coronavirus spike protein by human airway trypsin-like protease. J Virol. (2011) 85:13363–72. 10.1128/JVI.05300-11 21994442 PMC3233180

[24] Hoffmann M Kleine-Weber H Schroeder S Kruger N Herrler T Erichsen S. SARS-CoV-2 cell entry depends on ACE2 and TMPRSS2 and is blocked by a clinically proven protease inhibitor. Cell. (2020) 181:271–80.e8. 10.1016/j.cell.2020.02.052 32142651 PMC7102627

[25] Matsuyama S Nao N Shirato K Kawase M Saito S Takayama I. Enhanced isolation of SARS-CoV-2 by TMPRSS2-expressing cells. Proc Natl Acad Sci USA. (2020) 117:7001–3. 10.1073/pnas.2002589117 32165541 PMC7132130

[26] Lu G Hu Y Wang Q Qi J Gao F Li Y. Molecular basis of binding between novel human coronavirus MERS-CoV and its receptor CD26. Nature. (2013) 500:227–31. 10.1038/nature12328 23831647 PMC7095341

[27] Raj VS Mou H Smits SL Dekkers DH Muller MA Dijkman R. Dipeptidyl peptidase 4 is a functional receptor for the emerging human coronavirus-EMC. Nature. (2013) 495:251–4. 10.1038/nature12005 23486063 PMC7095326

[28] Walls AC Park YJ Tortorici MA Wall A McGuire AT Veesler D. Structure, function, and antigenicity of the SARS-CoV-2 spike glycoprotein. Cell. (2020) 181:281–92.e6. 10.1016/j.cell.2020.02.058 32155444 PMC7102599

[29] Kuba K Imai Y Rao S Gao H Guo F Guan B. A crucial role of angiotensin converting enzyme 2 (ACE2) in SARS coronavirus-induced lung injury. Nat Med. (2005) 11:875–9. 10.1038/nm1267 16007097 PMC7095783

[30] Yan R Zhang Y Li Y Xia L Guo Y Zhou Q. Structural basis for the recognition of the SARS-CoV-2 by full-length human ACE2. Science. (2020) 367:1444–8. 10.1126/science.abb2762 32132184 PMC7164635

[31] Hamming I Timens W Bulthuis ML Lely AT Navis G van Goor H. Tissue distribution of ACE2 protein, the functional receptor for SARS coronavirus. A first step in understanding SARS pathogenesis. J Pathol. (2004) 203:631–7. 10.1002/path.1570 15141377 PMC7167720

[32] Channappanavar R Perlman S. Pathogenic human coronavirus infections: causes and consequences of cytokine storm and immunopathology. Semin Immunopathol. (2017) 39:529–39. 10.1007/s00281-017-0629-x 28466096 PMC7079893

[33] Sims AC Baric RS Yount B Burkett SE Collins PL Pickles RJ. Severe acute respiratory syndrome coronavirus infection of human ciliated airway epithelia: role of ciliated cells in viral spread in the conducting airways of the lungs. J Virol. (2005) 79:15511–24. 10.1128/JVI.79.24.15511-15524.2005 16306622 PMC1316022

[34] Sims AC Burkett SE Yount B Pickles RJ SARS-CoV replication and pathogenesis in an *in vitro* model of the human conducting airway epithelium. Virus Res. (2008) 133:33–44. 10.1016/j.virusres.2007.03.013 17451829 PMC2384224

[35] Mossel EC Wang J Jeffers S Edeen KE Wang S Cosgrove GP. SARS-CoV replicates in primary human alveolar type II cell cultures but not in type I-like cells. Virology. (2008) 372:127–35. 10.1016/j.virol.2007.09.045 18022664 PMC2312501

[36] Qian Z Travanty EA Oko L Edeen K Berglund A Wang J. Innate immune response of human alveolar type II cells infected with severe acute respiratory syndrome-coronavirus. Am J Respir Cell Mol Biol. (2013) 48:742–8. 10.1165/rcmb.2012-0339OC 23418343 PMC3727876

[37] Harmer D Gilbert M Borman R Clark KL. Quantitative mRNA expression profiling of ACE 2, a novel homologue of angiotensin converting enzyme. FEBS Lett. (2002) 532:107–10. 10.1016/S0014-5793(02)03640-2 12459472

[38] Gu J Han B Wang J. COVID-19: gastrointestinal manifestations and potential fecal-oral transmission. Gastroenterology. (2020) 158:1518–9. 10.1053/j.gastro.2020.02.054 32142785 PMC7130192

[39] Lamers MM Beumer J van der Vaart J Knoops K Puschhof J Breugem TI. SARS-CoV-2 productively infects human gut enterocytes. Science. (2020). 10.1126/science.abc1669. [Epub ahead of print]. 32358202 PMC7199907

[40] Guan Y Zheng BJ He YQ Liu XL Zhuang ZX Cheung CL. Isolation and characterization of viruses related to the SARS coronavirus from animals in southern China. Science. (2003) 302:276–8. 10.1126/science.1087139 12958366

[41] Martina BE Haagmans BL Kuiken T Fouchier RA Rimmelzwaan GF Van Amerongen G. Virology: SARS virus infection of cats and ferrets. Nature. (2003) 425:915. 10.1038/425915a 14586458 PMC7094990

[42] Chen W Yan M Yang L Ding B He B Wang Y. SARS-associated coronavirus transmitted from human to pig. Emerg Infect Dis. (2005) 11:446–8. 10.3201/eid1103.040824 15757562 PMC3298239

[43] Shi J Wen Z Zhong G Yang H Wang C Huang B. Susceptibility of ferrets, cats, dogs, and other domesticated animals to SARS-coronavirus 2. Science. (2020). 10.1126/science.abb7015. [Epub ahead of print]. 32269068 PMC7164390

[44] Hatesuer B Bertram S Mehnert N Bahgat MM Nelson PS Pohlmann S. Tmprss2 is essential for influenza H1N1 virus pathogenesis in mice. PLoS Pathog. (2013) 9:e1003774. 10.1371/journal.ppat.1003774 24348248 PMC3857797

[45] Sakai K Ami Y Tahara M Kubota T Anraku M Abe M. The host protease TMPRSS2 plays a major role in *in vivo* replication of emerging H7N9 and seasonal influenza viruses. J Virol. (2014) 88:5608–16. 10.1128/JVI.03677-13 24600012 PMC4019123

[46] Tarnow C Engels G Arendt A Schwalm F Sediri H Preuss A. TMPRSS2 is a host factor that is essential for pneumotropism and pathogenicity of H7N9 influenza A virus in mice. J Virol. (2014) 88:4744–51. 10.1128/JVI.03799-13 24522916 PMC3993819

[47] Zmora P Blazejewska P Moldenhauer AS Welsch K Nehlmeier I Wu Q. DESC1 and MSPL activate influenza A viruses and emerging coronaviruses for host cell entry. J Virol. (2014) 88:12087–97. 10.1128/JVI.01427-14 25122802 PMC4178745

[48] Iwata-Yoshikawa N Okamura T Shimizu Y Hasegawa H Takeda M Nagata N. TMPRSS2 contributes to virus spread and immunopathology in the airways of murine models after coronavirus infection. J Virol. (2019) 93:e01815-18. 10.1128/JVI.01815-18 30626688 PMC6401451

[49] Li K Wohlford-Lenane CL Channappanavar R Park JE Earnest JT Bair TB. Mouse-adapted MERS coronavirus causes lethal lung disease in human DPP4 knockin mice. Proc Natl Acad Sci USA. (2017) 114:E3119–28. 10.1073/pnas.1619109114 28348219 PMC5393213

[50] Chow KT Gale MJr Loo YM. RIG-I and Other RNA Sensors in antiviral immunity. Annu Rev Immunol. (2018) 36:667–94. 10.1146/annurev-immunol-042617-053309 29677479

[51] Guillot L Le Goffic R Bloch S Escriou N Akira S Chignard M. Involvement of toll-like receptor 3 in the immune response of lung epithelial cells to double-stranded RNA and influenza A virus. J Biol Chem. (2005) 280:5571–80. 10.1074/jbc.M410592200 15579900

[52] Ioannidis I Ye F McNally B Willette M Flano E. Toll-like receptor expression and induction of type I and type III interferons in primary airway epithelial cells. J Virol. (2013) 87:3261–70. 10.1128/JVI.01956-12 23302870 PMC3592129

[53] Whitsett JA Alenghat T. Respiratory epithelial cells orchestrate pulmonary innate immunity. Nat Immunol. (2015) 16:27–35. 10.1038/ni.3045 25521682 PMC4318521

[54] Frieman M Heise M Baric R. SARS coronavirus and innate immunity. Virus Res. (2008) 133:101–12. 10.1016/j.virusres.2007.03.015 17451827 PMC2292640

[55] Wang Y Liu L. The membrane protein of severe acute respiratory syndrome coronavirus functions as a novel cytosolic pathogen-associated molecular pattern to promote beta interferon induction via a toll-like-receptor-related TRAF3-independent mechanism. MBio. (2016) 7:e01872–15. 10.1128/mBio.01872-15 26861016 PMC4752600

[56] Yoshikawa T Hill T Li K Peters CJ Tseng CT. Severe acute respiratory syndrome (SARS) coronavirus-induced lung epithelial cytokines exacerbate SARS pathogenesis by modulating intrinsic functions of monocyte-derived macrophages and dendritic cells. J Virol. (2009) 83:3039–48. 10.1128/JVI.01792-08 19004938 PMC2655569

[57] Chen IY Chang SC Wu HY Yu TC Wei WC Lin S. Upregulation of the chemokine (C-C motif) ligand 2 via a severe acute respiratory syndrome coronavirus spike-ACE2 signaling pathway. J Virol. (2010) 84:7703–12. 10.1128/JVI.02560-09 20484496 PMC2897593

[58] Alsafadi HN Uhl FE Pineda RH Bailey KE Rojas M Wagner DE. Applications and approaches for 3D precision-cut lung slices: disease modeling and drug discovery. Am J Respir Cell Mol Biol. (2020). 10.1165/rcmb.2019-0276TR. [Epub ahead of print]. 31991090 PMC7401444

[59] Wu W Booth JL Duggan ES Wu S Patel KB Coggeshall KM. Innate immune response to H3N2 and H1N1 influenza virus infection in a human lung organ culture model. Virology. (2010) 396:178–88. 10.1016/j.virol.2009.10.016 19913271 PMC2789846

[60] Delgado-Ortega M Melo S Punyadarsaniya D Rame C Olivier M Soubieux D. Innate immune response to a H3N2 subtype swine influenza virus in newborn porcine trachea cells, alveolar macrophages, and precision-cut lung slices. Vet Res. (2014) 45:42. 10.1186/1297-9716-45-42 24712747 PMC4021251

[61] Chokki M Yamamura S Eguchi H Masegi T Horiuchi H Tanabe H. Human airway trypsin-like protease increases mucin gene expression in airway epithelial cells. Am J Respir Cell Mol Biol. (2004) 30:470–8. 10.1165/rcmb.2003-0199OC 14500256

[62] Barbier D Garcia-Verdugo I Pothlichet J Khazen R Descamps D Rousseau K. Influenza A induces the major secreted airway mucin MUC5AC in a protease-EG, FR-extracellular regulated kinase-Sp1-dependent pathway. Am J Respir Cell Mol Biol. (2012) 47:149–57. 10.1165/rcmb.2011-0405OC 22383584

[63] Haga S Nagata N Okamura T Yamamoto N Sata T Yamamoto N. TACE antagonists blocking ACE2 shedding caused by the spike protein of SARS-CoV are candidate antiviral compounds. Antiviral Res. (2010) 85:551–5. 10.1016/j.antiviral.2009.12.001 19995578 PMC7114272

[64] Villeret B Dieu A Straube M Solhonne B Miklavc P Hamadi S. Silver nanoparticles impair retinoic acid-inducible gene i-mediated mitochondrial antiviral immunity by blocking the autophagic flux in lung epithelial cells. ACS Nano. (2018) 12:1188–202. 10.1021/acsnano.7b06934 29357226

[65] Teoh KT Siu YL Chan WL Schluter MA Liu CJ Peiris JS. The SARS coronavirus E protein interacts with PALS1 and alters tight junction formation and epithelial morphogenesis. Mol Biol Cell. (2010) 21:3838–52. 10.1091/mbc.e10-04-0338 20861307 PMC2982091

[66] Duan M Hibbs ML Chen W. The contributions of lung macrophage and monocyte heterogeneity to influenza pathogenesis. Immunol Cell Biol. (2017) 95:225–35. 10.1038/icb.2016.97 27670791

[67] Cheung CY Poon LL Ng IH Luk W Sia SF Wu MH. Cytokine responses in severe acute respiratory syndrome coronavirus-infected macrophages *in vitro*: possible relevance to pathogenesis. J Virol. (2005) 79:7819–26. 10.1128/JVI.79.12.7819-7826.2005 15919935 PMC1143636

[68] Law HK Cheung CY Ng HY Sia SF Chan YO Luk W. Chemokine up-regulation in SARS-coronavirus-infected, monocyte-derived human dendritic cells. Blood. (2005) 106:2366–74. 10.1182/blood-2004-10-4166 15860669 PMC1895271

[69] Dosch SF Mahajan SD Collins AR. SARS coronavirus spike protein-induced innate immune response occurs via activation of the NF-kappaB pathway in human monocyte macrophages *in vitro*. Virus Res. (2009) 142:19–27. 10.1016/j.virusres.2009.01.005 19185596 PMC2699111

[70] Al-Qahtani AA Lyroni K Aznaourova M Tseliou M Al-Anazi MR Al-Ahdal MN. Middle east respiratory syndrome corona virus spike glycoprotein suppresses macrophage responses via DPP4-mediated induction of IRAK-M and PPARgamma. Oncotarget. (2017) 8:9053–66. 10.18632/oncotarget.14754 28118607 PMC5354714

[71] Totura AL Whitmore A Agnihothram S Schafer A Katze MG Heise MT. Toll-like receptor 3 signaling via TRIF contributes to a protective innate immune response to severe acute respiratory syndrome coronavirus infection. MBio. (2015) 6:e00638–15. 10.1128/mBio.00638-15 26015500 PMC4447251

[72] Gralinski LE Menachery VD Morgan AP Totura AL Beall A Kocher J. Allelic variation in the toll-like receptor adaptor protein Ticam2 contributes to SARS-coronavirus pathogenesis in mice. G3. (2017) 7:1653–63. 10.1534/g3.117.041434 28592648 PMC5473747

[73] Sheahan T Morrison TE Funkhouser W Uematsu S Akira S Baric RS MyD88 is required for protection from lethal infection with a mouse-adapted SARS-CoV. PLoS Pathog. (2008) 4:e1000240 10.1371/journal.ppat.1000240 19079579 PMC2587915

[74] Cook DN Pisetsky DS Schwartz DA. Toll-like receptors in the pathogenesis of human disease. Nat Immunol. (2004) 5:975–9. 10.1038/ni1116 15454920

[75] Cao Y Li L Feng Z Wan S Huang P Sun X. Comparative genetic analysis of the novel coronavirus (2019-nCoV/SARS-CoV-2) receptor ACE2 in different populations. Cell Discov. (2020) 6:11. 10.1038/s41421-020-0147-1 32133153 PMC7040011

[76] Lim HK Huang SXL Chen J Kerner G Gilliaux O Bastard P. Severe influenza pneumonitis in children with inherited TLR3 deficiency. J Exp Med. (2019) 216:2038–56. 10.1084/jem.20181621 31217193 PMC6719423

[77] Channappanavar R Fehr AR Vijay R Mack M Zhao J Meyerholz DK. Dysregulated type I interferon and inflammatory monocyte-macrophage responses cause lethal pneumonia in SARS-CoV-infected mice. Cell Host Microbe. (2016) 19:181–93. 10.1016/j.chom.2016.01.007 26867177 PMC4752723

[78] Lee CT Fein AM Lippmann M Holtzman H Kimbel P Weinbaum G. Elastolytic activity in pulmonary lavage fluid from patients with adult respiratory-distress syndrome. N Engl J Med. (1981) 304:192–6. 10.1056/NEJM198101223040402 6969364

[79] Aschner Y Zemans RL Yamashita CM Downey GP. Matrix metalloproteinases and protein tyrosine kinases: potential novel targets in acute lung injury and ARDS. Chest. (2014) 146:1081–91. 10.1378/chest.14-0397 25287998 PMC4188143

[80] Sallenave JM Donnelly SC Grant IS Robertson C Gauldie J Haslett C. Secretory leukocyte proteinase inhibitor is preferentially increased in patients with acute respiratory distress syndrome. Eur Respir J. (1999) 13:1029–36. 10.1183/09031936.99.13510299 10414400

[81] Kido H Okumura Y Takahashi E Pan HY Wang S Yao D. Role of host cellular proteases in the pathogenesis of influenza and influenza-induced multiple organ failure. Biochim Biophys Acta. (2012) 1824:186–94. 10.1016/j.bbapap.2011.07.001 21801859

[82] Talmi-Frank D Altboum Z Solomonov I Udi Y Jaitin DA Klepfish M. Extracellular matrix proteolysis by MT1-MMP contributes to influenza-related tissue damage and mortality. Cell Host Microbe. (2016) 20:458–70. 10.1016/j.chom.2016.09.005 27736644

[83] Villeret B Solhonne B Straube M Lemaire F Cazes A Garcia-Verdugo I. Influenza a virus pre-infection exacerbates *Pseudomonas aeruginosa*-mediated lung damage through increased MMP-9 expression, decreased elafin production and tissue resilience. Front Immunol. (2020) 11:117. 10.3389/fimmu.2020.00117 32117268 PMC7031978

[84] Jiang Y Zhao G Song N Li P Chen Y Guo Y. Blockade of the C5a-C5aR axis alleviates lung damage in hDPP4-transgenic mice infected with MERS-CoV. Emerg Microbes Infect. (2018) 7:77. 10.1038/s41426-018-0063-8 29691378 PMC5915580

[85] Delgado-Ortega M Marc D Dupont J Trapp S Berri M Meurens F. SOCS proteins in infectious diseases of mammals. Vet Immunol Immunopathol. (2013) 151:1–19. 10.1016/j.vetimm.2012.11.008 23219158 PMC7112700

[86] Pothlichet J Chignard M Si-Tahar M. Cutting edge: innate immune response triggered by influenza A virus is negatively regulated by SOCS1 and SOCS3 through a RIG-I/IFNAR1-dependent pathway. J Immunol. (2008) 180:2034–8. 10.4049/jimmunol.180.4.2034 18250407

[87] Kedzierski L Linossi EM Kolesnik TB Day EB Bird NL Kile BT. Suppressor of cytokine signaling 4 (SOCS4) protects against severe cytokine storm and enhances viral clearance during influenza infection. PLoS Pathog. (2014) 10:e1004134. 10.1371/journal.ppat.1004134 24809749 PMC4014316

[88] Caly L Druce JD Catton MG Jans DA Wagstaff KM The FDA-approved Drug Ivermectin inhibits the replication of SARS-CoV-2 *in vitro*. Antiviral Res. (2020) 178:104787 10.1016/j.antiviral.2020.104787 32251768 PMC7129059

[89] Schrezenmeier E Dorner T. Mechanisms of action of hydroxychloroquine and chloroquine: implications for rheumatology. Nat Rev Rheumatol. (2020) 16:155–66. 10.1038/s41584-020-0372-x 32034323

[90] Mercuro NJ Yen CF Shim DJ Maher TR McCoy CM Zimetbaum PJ. Risk of QT interval prolongation associated with use of hydroxychloroquine with or without concomitant azithromycin among hospitalized patients testing positive for Coronavirus Disease 2019 (COVID-19). JAMA Cardiol. (2020). 10.1001/jamacardio.2020.1834. [Epub ahead of print]. 32936252 PMC7195692

[91] Amanat F Krammer F. SARS-CoV-2 vaccines: status report. Immunity. (2020) 52:583–9. 10.1016/j.immuni.2020.03.007 32259480 PMC7136867

[92] Jiang S Hillyer C Du L. Neutralizing antibodies against SARS-CoV-2 and other human coronaviruses. Trends Immunol. (2020) 41:355–9. 10.1016/j.it.2020.03.007 32249063 PMC7129017

[93] Gao Q Bao L Mao H Wang L Xu K Yang M. Rapid development of an inactivated vaccine candidate for SARS-CoV-2. Science. (2020) eabc1932. 10.1126/science.abc1932. [Epub ahead of print]. 32376603 PMC7202686

[94] Arts RJW Moorlag S Novakovic B Li Y Wang SY Oosting M. BCG vaccination protects against experimental viral infection in humans through the induction of cytokines associated with trained immunity. Cell Host Microbe. (2018) 23:89–100 e105. 10.1016/j.chom.2017.12.010 29324233

[95] Netea MG Joosten LAB. Trained immunity and local innate immune memory in the lung. Cell. (2018) 175:1463–5. 10.1016/j.cell.2018.11.007 30500533

[96] Yao Y Jeyanathan M Haddadi S Barra NG Vaseghi-Shanjani M Damjanovic D. Induction of autonomous memory alveolar macrophages requires t cell help and is critical to trained immunity. Cell. (2018) 175:1634–50 e1617. 10.1016/j.cell.2018.09.042 30433869

[97] Iwata-Yoshikawa N Uda A Suzuki T Tsunetsugu-Yokota Y Sato Y Morikawa S Effects of toll-like receptor stimulation on eosinophilic infiltration in lungs of BALB/c mice immunized with UV-inactivated severe acute respiratory syndrome-related coronavirus vaccine. J Virol. (2014) 88:8597–614. 10.1128/JVI.00983-14 24850731 PMC4135953

[98] Covián C Retamal-Díaz A Bueno SM Kalergis AM Could BCG vaccination induce protective trained immunity for SARS-CoV-2? Front Immunol. (2020) 11:970 10.3389/fimmu.2020.00970 PMC7227382 32574258

[99] Xing Z Afkhami S Bavananthasivam J Fritz DK D'Agostino MR Vaseghi-Shanjani M. Innate immune memory of tissue-resident macrophages and trained innate immunity: re-vamping vaccine concept and strategies. J Leukoc Biol. (2020). 10.1002/JLB.4MR0220-446R. [Epub ahead of print]. 32125045

[100] Sallenave JM Guillot L Host signaling and proteolytic pathways in the resolution or the exacerbation of coronavirus (CoV-2) infection in COVID-19 disease: what therapeutic targets? (2020) 10.31219/osf.io/rtfmx PMC7270404 32574272

